# Active learning for ordinal classification based on expected cost minimization

**DOI:** 10.1038/s41598-022-26844-1

**Published:** 2022-12-28

**Authors:** Deniu He

**Affiliations:** Chongqing Key Laboratory of Computational Intelligence, Chongqing University of Posts and Telecommunications, No. 2 Chongwen Road, Nan’an District, Chongqing, 400065 China

**Keywords:** Classification and taxonomy, Computational models, Data acquisition, Data mining, Machine learning

## Abstract

To date, a large number of active learning algorithms have been proposed, but active learning methods for ordinal classification are under-researched. For ordinal classification, there is a total ordering among the data classes, and it is natural that the cost of misclassifying an instance as an adjacent class should be lower than that of misclassifying it as a more disparate class. However, existing active learning algorithms typically do not consider the above ordering information in query selection. Thus, most of them do not perform satisfactorily in ordinal classification. This study proposes an active learning method for ordinal classification by considering the ordering information among classes. We design an expected cost minimization criterion that imbues the ordering information. Meanwhile, we incorporate it with an uncertainty sampling criterion to impose the query instance more informative. Furthermore, we introduce a candidate subset selection method based on the *k*-means algorithm to reduce the computational overhead led by the calculation of expected cost. Extensive experiments on nine public ordinal classification datasets demonstrate that the proposed method outperforms several baseline methods.

## Introduction

Ordinal classification (OC) is a particular case of multi-class classification task where the output variables come along with a natural total ordering, i.e., the instances are labeled by ordinal scales^1,2^. Since an ordered relation exists among the classes in many real situations, ordinal classification has a wide range of applications. For instance, clinical treatment^3–5^ in the medical field, bank failure prediction^6,7^ in the financial field, facial age estimation^8,9^ in the computer vision field, and so forth. As a supervised learning task, OC usually relies on a sufficient amount of labeled data to train an ordinal prediction model or induce the rules. However, the label acquisition for ordinal instances is usually expensive and time-consuming due to the dependence on human preference and domain expertise^10,11^, prohibiting the collection of a large number of labeled instances. In this situation, one can use the active learning (AL) technique^12–14^ to train an ordinal classifier^15,16^. Active learning aims to reduce the labeling cost by selectively labeling a small set of valuable instances. Therefore, the fundamental issue of an AL method is critical instance selection (also called query selection). The query selection strategy is usually designed based on an existing prediction model. In each iteration of an AL process, the query selection strategy is used to select the most valuable unlabeled instances. Then, the AL algorithm queries the labels of these instances and retrains a prediction model. This work aims to design an effective AL method for ordinal classification.

In the past few decades, many well-established multi-class AL methods have been designed, but little attention has been paid to the AL problem for ordinal classification. Existing multi-class AL methods usually perform unsatisfactorily in ordinal classification scenarios because they are typically designed for nominal multi-class classification problems. In ordinal classification, the cost of misclassifying an instance as an adjacent class should be lower than that of misclassifying it as a more disparate class^2,3,5^. We call this principle the ordering information among the ordinal classes. For example, in the financial field, customers’ credit scores can be categorized as “bad”, “fair”, “good”, and “excellent”^5^. It is clear that the cost or risk of misclassifying a “bad” customer as “excellent” is higher than misclassifying this customer as “fair”. Several studies have confirmed that the above ordering information between labels benefits constructing more accurate ordinal prediction models^1,2,17–20^. Such as the cost-sensitive ordinal classification models based on absolute or quadratic cost^1,17,19^.

In this paper, we introduce an expected cost minimization criterion that imbues the ordering information to guide critical instance selection in AL for ordinal classification. Therefore, we call our method active learning for ordinal classification based on expected cost minimization (abbreviated as AOCECM). Our method follows a one-step-look-ahead manner and chooses the instance that, if labeled, the base learner can obtain a minimal expected misclassification cost on the unlabeled instance set. We use the absolute misclassification cost to represent the ordering information and estimate the expected cost. Furthermore, to enforce the selected instance more informative, we integrate the expected cost minimization with a margin-based uncertainty sampling criterion. Thus, the critical instances can be selected in a complementary way. Our AL method employs the recently proposed kernel extreme learning machine-based ordinal classification model (KELMOR^1^) as the base learner. There are multiple models for ordinal classification in the literature (e.g., SVOR^21^, KDLOR^22^, and so on), and KELMOR is one of them. The KELMOR model is used as the base learner because it can achieve incremental updates and has competitive ordinal classification performance.

In our method, the calculation of expected cost is computationally intensive, which may lead the algorithm intractable in implementation. To mitigate this dilemma, we present a candidate subset selection method based on the *k*-means algorithm^23^ from a granular computing perspective. Granular computing usually follows a scheme of divide and conquer, thus making a complex problem simple and feasible^24^. By borrowing this idea, we divide the data into multiple granules with *k*-means clustering according to the number of labeled instances in each iteration of our active learning method. Thus, the instances can be divided into “described granules” and “undescribed granules”. If a granule contains labeled instances, we refer to it as a described granule. Conversely, if a granule only contains unlabeled instances, we call it an undescribed granule. It is known that the centroid point of a granule is generally representative of a granule. Moreover, the centroid points from different granules maintain the property of diversity. Therefore, in each iteration of our algorithm, we select the centroid point of the undescribed granules as the candidate instances. Conducting query selection in the candidate subset can substantially reduce the computational overhead and simultaneously endow the selected instances with the properties of representative and diversity.

For the sake of brevity, the main contributions of this work are summarized as follows.

- This paper proposes a novel active learning method for ordinal classification. We design an expected cost minimization criterion by considering the ordering information between ordinal classes. This criterion guides the algorithm to select the instances that are most likely to reduce the expected misclassification cost of the base learner. Moreover, we incorporate this criterion with an uncertainty sampling criterion to select valuable instances in a complementary way.
- We design a candidate subset selection method based on the *k*-means algorithm, which greatly reduces the computational overhead of calculating the expected cost and endows the selected instances with representative and diversity.
- Extensive experiments on nine public ordinal datasets demonstrate that the proposed method is superior to the competitors.

The remainder of this paper is organized as follows. Section 2 reviews the related work from the aspect of active learning and recalls the base learner used in our AL method. Section 3 provides the technical details of the proposed method. The experiment setting and experimental results are reported in Sect. 4. Finally, conclusions and future work are discussed in Sect. 5.

## Background

This section briefly reviews literature in the active learning field related to our work. In addition, we also recall the basic structure of the kernel extreme learning machine-based OC model^1^ because it is used as the base learner in our method.

### Related work

AL benefits many machine learning settings where a large amount of unlabeled data is available or easy to collect but labeling them is expensive, time-consuming, or exhausting. An active learner generally consists of a base learner (a prediction model) and a query selection strategy. The critical issue of the AL study is developing a query selection strategy to determine which candidate instances are most valuable if labeled. Traditional AL strategies mainly focus on assessing the informativeness or representativeness of candidate instances.

The AL strategies concerning instance’s informativeness include uncertainty sampling^25–27^, query by committee^28,29^, expected change^30–32^, and so on. Uncertainty sampling follows a confidence-estimation heuristic and selects the instance for which its current prediction is maximally uncertain^25^. In multi-class classification scenarios, the following three criteria are commonly utilized to measure uncertainty: (1) Least confidence^26^, which defines the most valuable instance as the one with the lowest maximum posterior estimate among all classes. (2) Margin-based sampling^27^, which selects the instance closest to the decision boundary or with the lowest discrepancy in its top two class predictions. (3) Maximum entropy^25^, which chooses the instance with the largest information entropy based on the posterior estimates over all classes. Although the uncertainty sampling methods are susceptible to selecting redundant instances and outliers, they are the most commonly used AL schemes and have been shown to work well^33^. Query-by-committee (QBC) trains a set of prediction models, and the unlabeled instances with the greatest disagreement in model decisions are selected^29^. This approach benefits from multiple classifiers providing different views of the input data^34^. The fundamental issue of the QBC scheme is how to quantify the disagreement to define a strategy to select the new instances. The QBC can apply to multi-class settings by employing multiple multi-class classification models, but a potential bias introduced by the induced models may limit its performance. The expected change-based AL scheme follows a decision-theoretic manner, which estimates the change in the model caused by an unlabeled instance being assigned to one of the possible labels and weights the change by an estimate of its probability^13^. This AL scheme includes expected model change^35^, expected error reduction^31^, expected performance change^32^, and so on. However, most expected change-based AL methods are computationally expensive. In this paper, to use the ordering information to guide the query selection, we borrow the expected change-based AL scheme to compute the expected cost minimization. Considering the prohibitive computational cost of this scheme, we design a candidate subset selection method to reduce the computational overhead significantly.

Representativeness-based AL strategy aims to select the instances that can represent the data distribution. The most frequently used methods of this type include experimental designs^15,36,37^ and clustering assumption-based AL methods^38–40^. The experimental design aims to minimize the model parameter variances by relying on a certain data reconstruction framework^41^. The clustering-based active learning methods explore the clustering or manifold structure of the data and select the instances that represent the intrinsic geometry of the data. Although the clustering-based AL approaches are suitable for multi-class classification AL tasks, their major drawback is that the performance depends on the quality of the clustering results^39^. Many regression-oriented AL methods prefer to consider the representativeness of candidate instances^42–44^. Active regression methods that do not rely on regression models usually select key instances by considering the diversity of instances, such as the methods in^42,43^. In an ordinal classification setting, informative instances are usually distributed between adjacent classes, but these regression-oriented methods fail to capture the informative instances in ordinal data. The regression AL methods that depend on regression models include experimental design-based methods^36^, expected model change-based methods^45^, and so on. Although ordinal classification is also referred to as ordinal regression, it is essentially a multi-class classification problem. In particular, ordinal classification models are typically specially designed. Therefore, these AL methods that rely on specific regression models usually perform unsatisfactorily for ordinal classification.

In the AL community, there is no doubt that AL methods that consider multiple query selection criteria typically perform better than those using only a single criterion. For instance, it has been suggested to incorporate the clustering techniques into conventional active learning strategies, thus providing complementary information for query selection^37,46^. In^47^, the authors combined the information density weight with an uncertainty sampling. While the study in^48^ has stated the importance of sampling diversity in uncertainty sampling. In this paper, we simultaneously consider the ordering information and uncertainty sampling-based informativeness in the query selection. In addition, the *k*-means-based adaptive candidate subset selection can impose our algorithm to select representative and diverse instances.

Although much progress has been made in AL algorithms^49,50^, little attention has been focused on ordinal classification. Soons and Feelders^51^ first build an AL method for ordinal classification, which selects instances by exploiting the monotonicity constraints in the data. But, this method is only applicable to monotonic classification problems^52^ and cannot scale up to the general ordinal classification problem. Xue and Hauskrecht^32^ proposed an AL method by querying ordinal scale labels, but this method is actually aimed at the active learning problems for binary classification. Recently, Li et al.^15^ introduced an A-optimal experimental design method for ordinal classification based on an adjacent category logistic model. However, this method needs to calculate the inverse of a large matrix. The prohibitive computational cost limits its usability in practice. In the imbalanced ordinal classification study, Ge et al.^16^ employed a margin-based uncertainty sampling strategy in ordinal classification to achieve oversampling. It is clear that this method is susceptible to the problems of uncertainty sampling, such as sampling redundancy, selecting outliers, and so on. To the best of our knowledge, the above two works are the only two AL methods in the context of ordinal classification. However, the above two methods fail to consider the ordering information in query selection. The above situation motivates this study to design a more effective AL method for ordinal classification.

### Ordinal classification based on kernel extreme learning machine

Our active learning approach employs the recently proposed kernel extreme learning machine-based OC model (KELMOR^1^) as the base learner. Thus, it is essential to recall it as preparatory knowledge briefly.

Given a training set $\{({\textbf{x}}_i,y_i)\}_{i=1}^{n}$, where ${\textbf{x}}_i \in {\mathbb {R}}^d$ denotes the *i*-th instances, *d* is the dimension of the data, $y_i \in {\mathcal{Y}}=\{{\mathcal{C}}_1,{\mathcal{C}}_2,\ldots ,{\mathcal{C}}_K\}$ is the label corresponding to ${\textbf{x}}_i$, and *K* is the number of classes. Compared with standard nominal multi-class classification, ordinal classification maintains an ordered relationship among the classes. Such as ${\mathcal{C}}_1< {\mathcal{C}}_2< \cdots < {\mathcal{C}}_K$, where the notation “<” represents a certain ordering relation or grading relation. In this context, ${\mathcal{C}}_k$ is only adjacent to ${\mathcal{C}}_{k-1}$ and ${\mathcal{C}}_{k+1}$. Generally, ordinal classification aims to learn a model that can map an unobserved instance to a label as close to the true label as possible.

The KELMOR model adopts an encoding-learning-predicting-decoding procedure. In the KELMOR model, each class label is firstly encoded based on a quadratic cost encoding scheme. Hence, the *k*-th class label is encoded as

1 $$\begin{aligned} {\textbf{t}}_k = [(1-k)^2, (2-k)^2, \ldots , (K-k)^2], \end{aligned}$$

Then, the training set of $\{({\textbf{x}}_i,y_i)\}_{i=1}^{n}$ is transformed into $\{({\textbf{x}}_i,{\textbf{y}}_i)\}_{i=1}^{n}$, where ${\textbf{y}}_i \in \{ {\textbf{t}}_1, \ldots , {\textbf{t}}_K\}$ is an encoded label vector. Thus, we obtain an encoded target matrix ${\textbf{T}}\in {\mathbb {R}}^{n\times K}$ concerning the training instances. The *i*-th row of ${\textbf{T}}$ is the encoded label vector of the training instance ${\textbf{x}}_i$. The benefit of using a quadratic cost encoding scheme is that it can imbue the ordering information between labels and enlarge the cost-sensitive distance.

In the learning phase, the KELMOR model learns a weight matrix ${\hat{\beta }} \in {\mathbb {R}}^{n \times K}$ that can project an unobserved instance from the feature space into a *K* dimensional output vector. The weight matrix ${\hat{\beta }}$ is computed as

2 $$\begin{aligned} {\hat{\beta }} = \left(\frac{1}{C}{\textbf{I}} + {\textbf{K}}\right)^{-1}{\textbf{T}}\,, \end{aligned}$$

where ${\textbf{I}} \in {\mathbb {R}}^{n\times n}$ is an identity matrix, *C* is a trade-off between the training error and the generalization ability, and ${\textbf{K}} \in {\mathbb {R}}^{n \times n}$ is a kernel matrix. The kernel matrix can be computed by using a certain kernel function ${\textbf{K}}_{ij} = {\mathcal{K}}({\textbf{x}}_i,{\textbf{x}}_j)$, such as the RBF kernel.

In the predicting phase, the predicted output of the KELMOR model for an unobserved instance ${\textbf{x}}$ is formulated as

3 $$\begin{aligned} \begin{aligned}{}&{\textbf{f}}({\textbf{x}}) = {\textbf{k}}({\textbf{x}}) {\hat{\beta }} \\&\quad \,\,\,\, ={\textbf{k}}({\textbf{x}})(\frac{1}{C}{\textbf{I}} + {\textbf{K}})^{-1}{\textbf{T}}, \end{aligned} \end{aligned}$$

where ${\textbf{k}}({\textbf{x}}) = [{\mathcal{K}}({\textbf{x}},{\textbf{x}}_{1}),{\mathcal{K}}({\textbf{x}},{\textbf{x}}_2),\ldots ,{\mathcal{K}}({\textbf{x}},{\textbf{x}}_n)]$, and ${\textbf{f}}({\textbf{x}}) \in {\mathbb {R}}^{K}$ is the predicted output.

To obtain ${\textbf{x}}$’s the ordinal scale label, the predicted output ${\textbf{f}}({\textbf{x}})$ should be decoded as follows

4 $$\begin{aligned} {\hat{y}} = \mathop {\arg \min }\limits _{{\mathcal{C}}_k\in \{{\mathcal{C}}_1,\ldots ,{\mathcal{C}}_K\}} \left\Vert {\textbf{f}}({\textbf{x}}) - {\textbf{t}}_{k}\right\Vert _{1} \,, \end{aligned}$$

where $\left\Vert \cdot \right\Vert _{1}$ denotes the $l_1$-norm of a vector, ${\textbf{t}}_{k}$ is the encoding label vector that corresponds to the *k*-th class. Eq. (4) is referred to as the decoding process. For more details about the KELMOR model, readers can refer to reference^1^. The time complexity of training a KELMOR model is cubic with the number of training instances. In Sect. 3.4, we will introduce how to update the KELMOR model incrementally. Therefore, we can incrementally retrain the KELMOR model when a newly observed instance is added to the training set. The time complexity of incrementally retraining the KELMOR model is quadratic with the number of training instances.

## The proposed method

### Method overview

**Figure 1:**
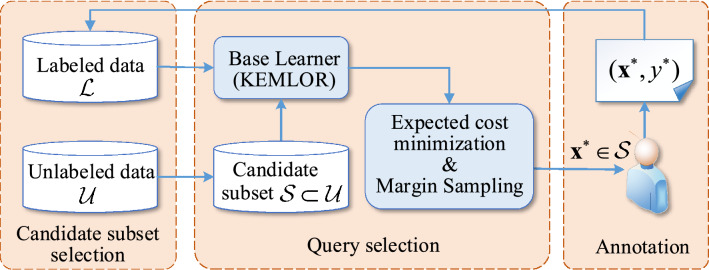
Framework of the proposed method.

The framework of the proposed method is depicted in Fig. 1. In the considered AL setting, let ${\mathcal{L}}=\{({\textbf{x}}_i,y_i)\}_{i=1}^{n}$ be the initial training set and ${\mathcal{U}}=\{{\textbf{x}}_i\}_{i=n+1}^{N}$ be the pool set. Our AL method consists of two main components. One component is candidate subset selection, and the other is query selection. In each iteration, our method selects a set of candidate instances ${\mathcal{S}}$ from the unlabeled pool ${\mathcal{U}}$; then, a query instance is selected from ${\mathcal{S}}$ to query the annotator. After the query instance and its label are added to ${\mathcal{L}}$, we retrain the base learner. The above process is repeated until the given query budget is exhausted.

The candidate subset ${\mathcal{S}}$ in each iteration is selected based on a *k*-means clustering-based candidate subset selection method. The query selection strategy is designed by integrating an expected cost minimization criterion and a margin sampling criterion. The ordering information between classes is imbued in the expected cost minimization criterion. Since the candidate subset selection serves the query selection, we will first describe the query selection method in the following subsections.

### Query selection

In the context of ordinal classification, a prediction model mainly focuses on minimizing the misclassification cost in prediction by considering the ordering information among classes. Inspired by this, we design an expected cost minimization criterion to select candidate instances that, if labeled, can minimize the base learner’s misclassification cost on the unlabeled instances. We use the absolute misclassification cost to calculate the expected cost. Thus, the ordering information is imbued into the query selection.

According to the above idea, we can calculate the expected cost of the KELMOR model for each unlabeled instance in a one-step-look-ahead manner. Given a training set ${\mathcal{L}}$, denote by $P_{{\mathcal{L}}}({\mathcal{C}}_{k} |{\textbf{x}})$ the probability estimate for a particular candidate instance ${\textbf{x}} \in {\mathcal{U}}$ based on the KELMOR model, where $k=1,\ldots ,K$. Suppose the candidate instance ${\textbf{x}}$ is assigned a possible label ${\mathcal{C}}_k$ and added into the training set. We use $P_{{\mathcal{L}}\cup \{({\textbf{x}},{\mathcal{C}}_k)\}}({\mathcal{C}}_r |{\textbf{x}}_i)$ to denote the probability estimate for an unlabeled instance ${\textbf{x}}_i \in {\mathcal{U}}/\{{\textbf{x}}\}$ with the KELMOR model trained on ${\mathcal{L}}\cup \{({\textbf{x}},{\mathcal{C}}_k)\}$, where $r=1,\ldots ,K$. Thus, the expected cost by labeling ${\textbf{x}} \in {\mathcal{U}}$ can be defined as

5 $$\begin{aligned} EC({\textbf{x}}) = \sum \limits _{k=1}^{K}P_{{\mathcal{L}}}({\mathcal{C}}_{k} |{\textbf{x}}) \frac{1}{|{\mathcal{U}}/\{{\textbf{x}}\}|}\sum \limits _{i=1}^{|{\mathcal{U}}/\{{\textbf{x}}\} |} \sum \limits _{r=1}^{K} P_{{\mathcal{L}}\cup \{({\textbf{x}},{\mathcal{C}}_k)\}}({\mathcal{C}}_{r} |{\textbf{x}}_{i}){\textbf{C}}_{hr} \end{aligned}$$

where ${\textbf{C}}_{hr} = \left|h-r \right|$ is the absolute misclassification cost and $h = \mathop {\arg \max }\limits _{r\in \{1,\ldots ,K\}} P_{{\mathcal{L}} \cup \{({\textbf{x}},{\mathcal{C}}_k)\}}({\mathcal{C}}_r|{\textbf{x}}_i)$, which means ${\textbf{x}}_i$ has the highest probability estimate at the *h*-th class. Ideally, the misclassification cost should be determined based on a priori knowledge. However, in most cases, a priori knowledge does not exist^2^. Therefore, we use the absolute cost as the proxy for the ordering information among classes. According to the principle of the expected cost minimization, we can determine the critical instances as follows

6 $$\begin{aligned} {\textbf{x}}^{*} = \mathop {\arg \min }\limits _{{\textbf{x}}\in {\mathcal{U}}} EC({\textbf{x}}) \,. \end{aligned}$$

To fully use of the available information and make the query selection more effective, we combine a margin-based uncertainty sampling criterion with the expected cost minimization criterion. In ordinal classification data, the informative instances are usually distributed in the regions between adjacent classes. The margin-based sampling criterion tends to query instances in those regions. By introducing the margin-based sampling criterion, the expected cost minimization criterion can be promoted to select valuable instances close to the decision boundaries, which benefits quickly improving the prediction model. Besides, the margin-based sampling runs fast and its computational cost is almost negligible compared with the expected cost minimization. Given a candidate instance ${\textbf{x}} \in {\mathcal{U}}$, the margin sampling criterion is computed as

7 $$\begin{aligned} MS({\textbf{x}}) = P_{{\mathcal{L}}}({\hat{y}}^{1} |{\textbf{x}}) - P_{{\mathcal{L}}}({\hat{y}}^{2}|{\textbf{x}}) \,, \end{aligned}$$

where ${\hat{y}}^{1}$ and ${\hat{y}}^{2}$ are the first and second most likely predictive labels about instance ${\textbf{x}}$. The margin sampling chooses the instance with the minimum value of $MS({\textbf{x}})$. Therefore, to simultaneously consider the above two criteria, we define the acquisition function as

8 $$\begin{aligned} {\textbf{x}}^{*} = \mathop {\arg \min }\limits _{{\textbf{x}}\in {\mathcal{U}}} \lambda EC({\textbf{x}}) + (1-\lambda ) MS({\textbf{x}}), \end{aligned}$$

where $0 \le \lambda \le 1$ is a constant which controls the contributions of expected cost minimization and uncertainty sampling criteria.

The calculation of expected cost relies on the probability estimate of class membership for the unlabeled instances. However, the KELMOR model does not yield the probability estimate. Therefore, We design a method based on the softmax function to obtain the probability estimate. We define $NR({\mathcal{C}}_k |{\textbf{x}})= \Vert {\textbf{f}}({\textbf{x}}) - {\textbf{t}}_{k}\Vert _{1}$ as the rejection degree of ${\textbf{x}}$ belongs to class ${\mathcal{C}}_k$, where ${\textbf{f}}({\textbf{x}})$ is the predicted output vector of the KELMOR model, and ${\textbf{t}}_k$ is the encoded label vector of the *k*-th class label. Thus, the probability estimate about instance ${\textbf{x}}$ can be defined as

9 $$\begin{aligned} P({\mathcal{C}}_k |{\textbf{x}}) = \frac{e^{-NR({\mathcal{C}}_k|{\textbf{x}})}}{\sum \limits _{j=1}^{K} e^{-NR({\mathcal{C}}_j |{\textbf{x}})}}, \quad k=1,\ldots ,K\,. \end{aligned}$$

According to Eq. (5), we can see that the calculation of expected cost for all the unlabeled instances is computationally intensive. Not only does it require computing the misclassification cost over ${\mathcal{U}}$ for each unlabeled instance, but the KELMOR model should be retrained by adding each possible query instance with all possible labels into the training set. The time complexity for calculating the expected cost for all the unlabeled instances is ${\mathcal{O}}(\left|{\mathcal{U}} \right|\cdot \left|{\mathcal{L}}\right|^3 + \left|{\mathcal{U}} \right|^2\cdot \left|{\mathcal{L}} \right|\cdot K)$. To reduce the computational cost of query selection, we shall reduce the candidate set in each iteration. Therefore, we introduce a candidate subset selection method in the next subsection.

### Candidate subset selection

To reduce the computational overhead of the above query selection, we design an adaptive candidate subset selected method based on the *k*-means algorithm^23^.

Before commencing a query selection, we first perform *k*-means algorithm on the whole instances ${\mathcal{L}}\cup {\mathcal{U}}$ and cluster them into $(|{\mathcal{L}} |+1)$ granules. Therefore, there will be at least one granule that does not contain any labeled instances. Then, the centroids of granules that do not contain any labeled instances are collected as the candidate subset. As we mentioned before, we refer to those granules that do not contain any labeled instances as undescribed granules. In practice, some granules may contain more than one labeled instance; thus, there is usually more than one undescribed granule. The *k*-means algorithm is employed because of its low computational cost. In addition, it typically produces spherical shape granules with relatively uniform sizes^53^. Since candidate instances come from the centers of different spherical granules, they are typically diverse and representative.

**Figure 2:**
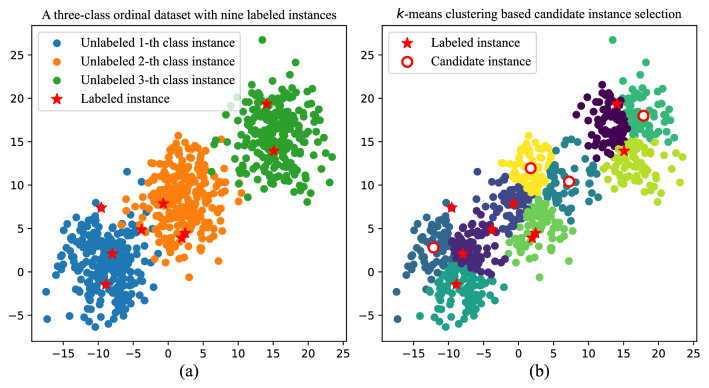
Example of candidate subset selection. Subfigure (**a**) shows a three-class synthetic ordinal dataset of 800 instances, with 9 labeled instances in the current iteration. To obtain a candidate subset, we use the *k*-means algorithm to divide the data into 10 clusters. Then, we take the centroids of clusters containing no labeled instances as candidate instances. Subfigure (**b**) shows the result of candidate subset selection. We can see that there are 4 candidate instances in the current iteration.

Figure 2 shows an example of candidate subset selection on a three-class synthetic ordinal dataset, which currently includes 9 labeled and 791 unlabeled instances. According to the above description, we need to divide the 800 instances into 10 granules by performing the *k*-means algorithm. Then, we obtain 4 undescribed granules. Therefore, the current candidate subset contains 4 representative instances. Consequently, in the current iteration, we only need to calculate the margin sampling criterion and the expected cost minimization criterion on the 4 candidate instances rather than on the 791 unlabeled instances. Taking into account the cost of clustering and finding a candidate subset, it is computationally more cost-effective to perform query selection by first finding a subset of candidates than by performing query selection directly on the unlabeled instance set. We will discuss the time complexity of the proposed method in Sect. 3.5.

### KELMOR model incremental update

In the active learning process, we must retrain the KELMOR model after each new instance is added to the training set. As aforementioned, the time complexity of training a KELMOR model is cubic with the number of training instances. This subsection introduces an incremental update method for the KELMOR model. Based on the incremental update method, the time complexity of incrementally retraining a KELMOR model is quadratic with the number of training instances.

Suppose there are *n* instances in the current training set ${\mathcal{L}}$. Let ${\textbf{T}}$ and ${\textbf{K}}$ be the encoded target matrix and kernel matrix corresponding to ${\mathcal{L}}$, respectively. When a new instance ${\textbf{x}}^{*}$ is labeled, and its encoded label vector is ${\textbf{t}}^{*}$, the expanded training set becomes $\bar{{\mathcal{L}}} = {\mathcal{L}} \cup \{({\textbf{x}}^{*}, {\textbf{t}}^{*})\}$. Thus, the new weight matrix of the KELMOR model can be formulated as

10 $$\begin{aligned} \begin{aligned} {\bar{\beta }}&= (\bar{{\textbf{K}}}+\frac{1}{C}{\textbf{I}}_{n+1})^{-1} \begin{bmatrix} {\textbf{T}} \\ {\textbf{t}}^{*} \end{bmatrix} \\&= \begin{bmatrix} {\textbf{K}} + \frac{1}{C} {\textbf{I}}_n &{} {\textbf{k}}({\textbf{x}}^{*})\\ {\textbf{k}}({\textbf{x}}^{*})^{T} &{} {\mathcal{K}}({\textbf{x}}^{*},{\textbf{x}}^{*})+\frac{1}{C} \end{bmatrix}^{-1} \begin{bmatrix} {\textbf{T}} \\ {\textbf{t}}^{*} \end{bmatrix} \,, \end{aligned} \end{aligned}$$

where ${\textbf{k}}({\textbf{x}}^{*}) = [{\mathcal{K}}({\textbf{x}}_{1},{\textbf{x}}^{*}),\ldots ,{\mathcal{K}}({\textbf{x}}_{n},{\textbf{x}}^{*})]$ and ${\mathcal{K}}(\cdot ,\cdot )$ is a kernel function.

The time complexity of directly computing ${\bar{\beta }}$ is ${\mathcal{O}}((n+1)^3)$. Since $({\textbf{K}} + \frac{1}{C} {\textbf{I}}_n)^{-1}$ is available, we can compute ${\bar{\beta }}$ based on the block matrix inversion principle^54^. For conciseness, we reformulate ${\bar{\beta }}$ as:

11 $$\begin{aligned} \begin{aligned} {\bar{\beta }}&= \begin{bmatrix} {\textbf{A}}_{11} &{} {\textbf{A}}_{12}\\ {\textbf{A}}^{T}_{12} &{} {\textbf{A}}_{22} \end{bmatrix}^{-1} \begin{bmatrix} {\textbf{T}} \\ {\textbf{t}}^{*} \end{bmatrix} \,, \end{aligned} \end{aligned}$$

where

12 $$\begin{aligned} \begin{aligned}{}&{\textbf{A}}_{11} = {\textbf{K}} + \frac{1}{C} {\textbf{I}}_n \,,\\&{\textbf{A}}_{12} = {\textbf{k}}({\textbf{x}}^{*}) \,, \\&{\textbf{A}}_{22} = {\mathcal{K}}({\textbf{x}}^{*},{\textbf{x}}^{*})+\frac{1}{C} \,. \end{aligned} \end{aligned}$$

According to the block matrix inversion principle, the updated model can be represented as:

13 $$\begin{aligned} {\bar{\beta }} = \begin{bmatrix} {\textbf{B}}_{11} &{} {\textbf{B}}_{12}\\ {\textbf{B}}_{21} &{} {\textbf{B}}_{22} \end{bmatrix} \begin{bmatrix} {\textbf{T}} \\ {\textbf{t}}^{*} \end{bmatrix} \,, \end{aligned}$$

where

14 $$\begin{aligned} \begin{aligned}{}&{\textbf{B}}_{11} = A^{-1}_{11} + A^{-1}_{11}A_{12}(A_{22}-A^{T}_{12}A^{-1}_{11}A_{12})^{-1}A^{T}_{12}A_{11}^{-1} \,,\\&{\textbf{B}}_{12} = -A^{-1}_{11}A_{12}(A_{22}-A^{T}_{12}A^{-1}_{11}A_{12})^{-1} \,, \\&{\textbf{B}}_{21} = -(A_{22}-A^{T}_{12}A^{-1}_{11}A_{12})^{-1}A^{T}_{12}A^{-1}_{11} \,, \\&{\textbf{B}}_{22} = (A_{22}-A^{T}_{12}A^{-1}_{11}A_{12})^{-1} \,. \end{aligned} \end{aligned}$$

Suppose $K \ll n$, based on the above formulations, the computational complexity of calculating ${\bar{\beta }}$ is therefore reduced to ${\mathcal{O}}((n+1)^2K)={\mathcal{O}}(n^2)$.

### Algorithm and time complexity analyses

The algorithmic procedure of the proposed active learning method is summarized in Algorithm 1.
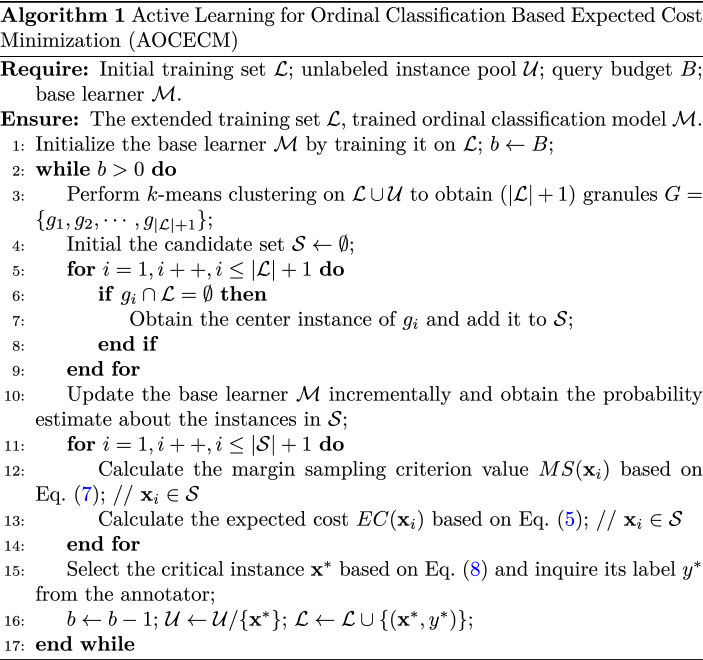


Suppose *N* is the number of all instances, *n* is the number of current labeled instances, and has $n \ll N$. In the pseudocode, lines 3 to 9 correspond to the procedure of candidate subset selection. Performing the *k*-means with $k=n+1$ requires ${\mathcal{O}}(N(n+1)t)$ time, where *t* denotes the number of iterations. Finding the undescribed granules in the worst situation requires ${\mathcal{O}}(Nn)$ time. Searching the representative point in undescribed granules in the worst situation requires ${\mathcal{O}}(\frac{Nn}{n+1})$ time. In summary, the time complexity of candidate subset selection is ${\mathcal{O}}(N(n+1)t)$. Line 10 to line 16 correspond to the procedure of query selection. Suppose we encounter the worst situation, i.e., there are $|{\mathcal{S}} |=n$ candidate instances in the current iteration. Update the KELMOR model incrementally in line 10 takes ${\mathcal{O}}(K(n+1)^2)$ time. Suppose the kernel matrix is pre-calculated. Thus, in line 12, calculating the margin sampling criterion for the *n* candidate instances takes ${\mathcal{O}}(n\log {K})$ time, where *K* is the number of classes. In line 13, the main cost of calculating the expected cost is the $(n\times K)$ times of re-training the KELMOR model, which requires ${\mathcal{O}}(nK^2(n+1)^2)$ time. In summary, the time complexity of the proposed method for one query selection in the worst situation is ${\mathcal{O}}(N(n+1)t + nK^2(n+1)^2)$.

In the case without the procedure of candidate instance selection, the time complexity of the algorithm will become ${\mathcal{O}}((N-n)K^2(n+1)^2)$. According to the above analysis, we can conclude that the proposed method will be more efficient than the case without candidate subset selection if the number of clustering iterations *t* satisfies the following condition:

15 $$\begin{aligned} t < \frac{(N-2n)K^2(n+1)}{N} \thickapprox K^2(n+1) \,. \end{aligned}$$

In ordinal classification, the number of classes *K* is typically equal to or larger than three. In an active learning setting, there is usually at least *K* labeled instance at the initial moment, and the number of labeled instances is increasing. Therefore, the inequality in Eq. (15) usually holds in practice. It is worth pointing out that the clustering results can be pre-calculated before active learning. From this point of view, the candidate subset selection brings an undeniable advantage in terms of computational time.

## Experiments

### Datasets

In the experiments, nine public ordinal classification datasets are employed. Table 1 summarizes the information of the used datasets. The datasets Thyroid, Knowledge, and Obesity are from the UCI machine learning repository. The other six datasets are from reference^2^. Before experiments, all the datasets are standardized by the following Z-score standardization:

16 $$\begin{aligned} x_{ij} = \frac{x_{ij}-mean(x_j)}{std(x_j)}, \end{aligned}$$

where $x_{ij}$ denotes the *j*-th attribute value of instance ${\textbf{x}}_i$, and $mean(x_j)$ and $std(x_j)$ are the mean value and the standard deviation of the *j*-th attribute, respectively.

**Table 1:** Information of the used datasets.

No.	Datasets	#Instances	#Features	#Classes	Distribution
1	Newthyroid^2^	215	5	3	[30, 150, 35]
2	Balance-scale^2^	625	4	3	[288, 49, 288]
3	Thyroid$^{1}$	7000	6	3	[166, 368, 6666]
4	Knowledge$^{1}$	403	5	4	[50, 129, 122, 102]
5	Machine^2^	209	6	5	[42, 42, 42, 42, 41]
6	Housing^2^	506	13	5	[102, 101, 101, 101, 101]
7	Computer^2^	8192	12	5	[1639, 1639, 1638, 1638, 1638]
8	Obesity$^{1}$	2111	16	7	[272, 287, 290, 290, 351, 297, 324]
9	Stock^2^	950	9	10	[95, 95, 95, 95, 95, 95, 95, 95, 95, 95]

https://archive.ics.uci.edu/ml/index.php.

### Experimental configurations

To validate the effectiveness of the proposed method AOCECM, we compare it with the following eleven state-of-the-art baseline methods.

- **Random** is the random sampling method. This method chooses the query instances randomly from the pool set. Therefore, it is also referred to as passive learning.
- **USME** is the uncertainty sampling method based on the KELMOR model and the entropy maximization strategy^25^.
- **USLC** is the uncertainty sampling method based on the KELMOR model and the least confidence strategy^26^.
- **USMS** is the uncertainty sampling method based on the KELMOR model and the margin-based sampling strategy^26,55^.
- **MCSVMA**^50^ is the SVM-based multi-class active learning method, which selects the instances by considering the criteria of rejection, compatibility, and uncertainty.
- **McPAL**^49^ is the multi-class probabilistic active learning method, which selects the instances with maximal probabilistic gain.
- **iGS**^44^ is an improved greedy sampling-based AL method. This method selects unlabeled instances to increase the diversity in both input and output spaces.
- **FISTA**^41^ is an extended transductive experimental design method based on an exclusive sparsity norm.
- **ALCE**^56^ is a multi-class active learning algorithm based on a cost embedding approach.
- **LogitA**^15^ is the A-optimal experimental design method for ordinal classification, which tends to query representative instances.
- **ALOR**^16^ is an uncertainty sampling-based AL method for ordinal classification based on the REDSVM model^57^. This method queries the instance with the smallest distance to the nearest separating hyperplane in each iteration.

In the experiment, each dataset is split by using the five-fold stratified cross-validation six times. Thus, there are a total of 30 splits, and each split corresponding to an independent experiment. In each split, a dataset is split into an unlabeled pool (80% of the data) and a testing set (20% of the data). The initial training set contains instances randomly selected one from each class in the unlabeled pool. The AL methods perform query selection in the unlabeled pool, and tested on the testing set. Finally, we report the average results of 30 runs. We simulate the annotator to provide the ground-truth labels of selected instances. The query budget for each dataset is set as 20*K*, where *K* is the number of classes.

In each iteration of active learning, we use labeled instances to train a KELMOR model and a REDSVM model^57^. We evaluate the ordinal classification performances of the two models on the testing set and record the average evaluation result. The parameter *C* in the KELMOR is fixed as 100. The kernel function ${\mathcal{K}}(\cdot ,\cdot )$ is set as the RBF kernel, and the $\gamma$ in the kernel function is set as 0.1 for all the datasets. For the trade-off parameter $\lambda$, we tune it from $[0.1,0.2,\ldots ,1.0]$ and report the best results. The evaluation metrics involve the Mean Zero-one Error (MZE), Mean Absolute Error (MAE), and Mutual Information (MI). The metrics MZE and MAE are longstanding benchmark metrics for ordinal classification^2^, while MI is a classical metric used to evaluate classification performance^58^. MZE denotes the error rate of a classifier:

17 $$\begin{aligned} MZE = \frac{1}{N_t}\sum \limits _{i=1}^{N_t}I[{\hat{y}}_i\ne y_i ] , \end{aligned}$$

where $y_i$ is the true label, $\hat{y_i}$ is the predicted label, and $N_t$ is the number of instances in the testing set. $I[\cdot ]$ is an indicator function that returns 1 if the argument is true and 0 otherwise. MZE considers a zero-one cost for misclassification. The MAE represents the average deviation in the absolute value of the predicted rank ${\mathcal{R}}({\hat{y}}_i)$ from the true one ${\mathcal{R}}(y_i)$:

18 $$\begin{aligned} MAE = \frac{1}{N_t}\sum \limits _{i=1}^{N_t}|{\mathcal{R}}({\hat{y}}_i) - {\mathcal{R}}(y_i)|, \end{aligned}$$

The MAE uses the absolute cost by considering the order between classes. Mutual information is used to measure the degree of coincidence between the true labels and the predicted labels, and which is formalized as follows:

19 $$\begin{aligned} MI = \sum \limits _{i=1}^{K}\sum \limits _{j=1}^{K}p_{ij}\log \left(\frac{p_{ij}}{p_{i}\times p_j}\right), \end{aligned}$$

where $p_{ij} = \frac{|\{{\textbf{x}}\in {\mathcal{T}}|y={\mathcal{C}}_i\} \cap \{{\textbf{x}}\in {\mathcal{T}}|{\hat{y}}={\mathcal{C}}_j\}|}{N_t}$, $p_{i}=\frac{|\{{\textbf{x}}\in {\mathcal{T}} |y={\mathcal{C}}_i\} |}{N_{t}}$, $p_{j}=\frac{|\{{\textbf{x}}\in {\mathcal{T}}|{\hat{y}}={\mathcal{C}}_j\}|}{N_{t}}$, and ${\mathcal{T}}$ is the testing set. Unlike the previous two metrics, the higher the value of MI, the better the classification performance.

To quantitatively compare the different methods, the commonly used metric Area Under Learning Curve (AULC)^59^ is employed. Let *B* be the query budget and $\pi$ be a particular classification performance metric. Thus, the AULC about $\pi$ is computed with the following trapezoidal approximation:

20 $$\begin{aligned} AULC = \sum \limits _{i=1}^{B} \pi (i), \end{aligned}$$

where $\pi (i)$ denotes the value of the metric $\pi$ in the *i*-th iteration. In the experiments, we will report the results of AULC about MZE (AULC-MZE), AULC about MAE (AULC-MAE), and AULC about MI (AULC-MI), respectively. In general, the lower the value of AULC-MZE and AULC-MAE, the better the performance of the AL algorithm. In contrast, the larger the value of AULC-MI, the better performance of the AL algorithm.

The experiments were implemented on Windows 10 64-bit operating system with 32GB RAM and an Intel(R) Core(TM) i7-8700 CPU@3.20GHz processor. The programming language is Python. The implementation of McPAL and ALCE relies on the active learning tool scikit-activeml^60^. The source codes are available at https://github.com/DeniuHe/AOCECM.

### Experimental result

**Figure 3:**
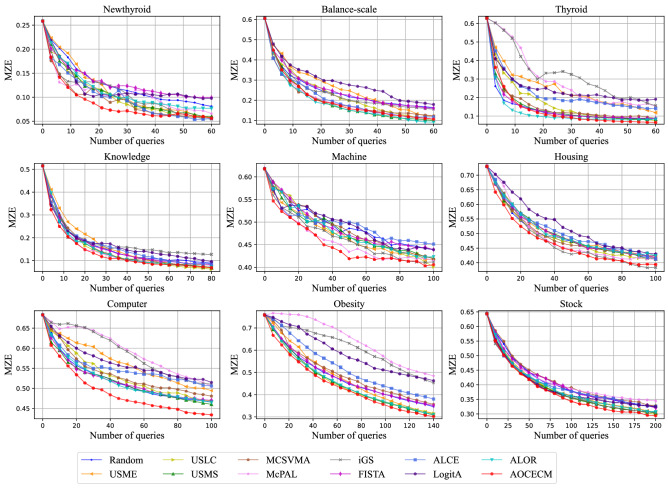
Learning curves of MZE for the twelve compared methods.

**Figure 4:**
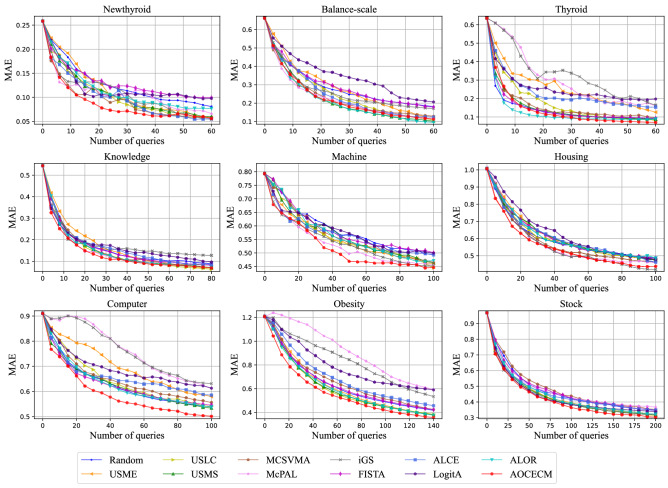
Learning curves of MAE for the twelve compared methods.

**Figure 5:**
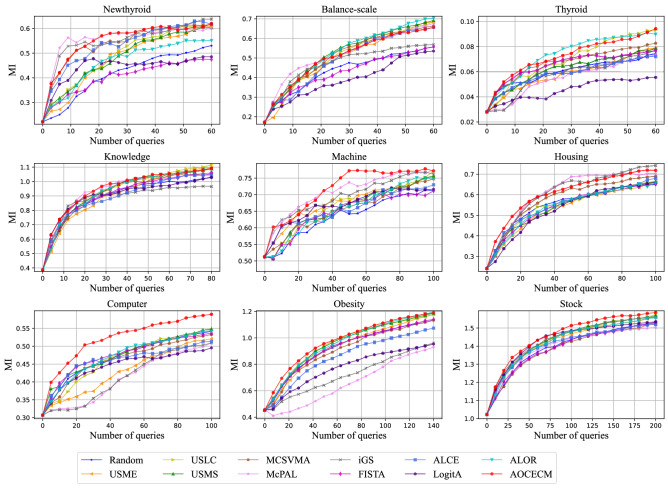
Learning curves of MI for the twelve compared methods.

To visually compare the proposed method with the eleven baseline methods, we plot the learning curves of the different methods on metrics MZE, MAE, and MI in Figs. 3, 4, and 5, respectively. In the above three figures, some learning curves inevitably overlap or cross since the comparison involves multiple compared methods. But, we can still clearly observe that the proposed method outperforms other methods in terms of the three metrics on most data sets.

For quantitative comparison, we report the evaluation results of the twelve methods on metrics AULC-MZE, AULC-MAE, and AULC-MI in Table 2. The best results are highlighted in boldface. We also show the average rank (denoted as “*AvgRank*”) of the compared methods in Table 2. To detect whether a baseline method performs significantly different from the AOCECM, we perform the Wilcoxon signed-rank test^61^ between the AOCECM and the baseline methods at a confidence level of $\alpha =0.05$. The marker “$*$” denotes that there is a statistically significant difference. To present the above statistical results more clearly, we summarize the win/tie/loss counts of the proposed method versus the baseline methods base on the Wilcoxon signed-rank test in Table 3. A win (or loss) is recorded when the proposed method is significantly better (or worse) than the compared method on a dataset in the Wilcoxon signed-rank test; otherwise, a tie is counted.

**Table 2:** Results of AULC-MZE , AULC-MAE, and AULC-MI for the twelve compared methods on the nine datasets.

Metric	Dataset	Random	USME	USLC	USMS	MCSVMA	McPAL	iGS	FISTA	ALCE	LogitA	ALOR	AOCECM
AULC-MZE	Newthyroid	7.82±2.85$*$	7.08±3.02$*$	6.43±2.64$*$	6.57±2.72$*$	5.82±3.00	6.71±3.90$*$	6.69±3.40$*$	8.07±2.68$*$	6.23±3.00$*$	7.51±3.08$*$	6.98±2.63$*$	**5.38±2.83**
	Balance-scale	15.24±3.33$*$	16.03±3.35$*$	14.01±3.18$*$	12.19±3.14	12.56±2.87	12.55±2.36	14.94±2.58$*$	15.45±3.13$*$	12.68±2.65	17.83±5.06$*$	**11.79±2.70**	12.43±2.65
	Thyroid	8.21±2.53	15.17±8.50$*$	10.55±5.32$*$	8.79±2.66	9.60±3.82$*$	18.03±10.09$*$	19.95±11.06$*$	8.43±2.42	13.55±8.01$*$	15.20±10.51$*$	**7.42±2.54**$*$	8.17±2.89
	Knowledge	13.40±3.87$*$	13.58±3.84$*$	11.90±3.60	12.18±3.52$*$	12.56±3.74$*$	11.84±3.58	14.60±3.52$*$	12.95±3.69$*$	13.36±3.92$*$	14.15±3.97$*$	11.99±3.38$*$	**11.14±3.12**
	Machine	49.85±6.02$*$	48.01±6.64$*$	48.68±7.18$*$	48.86±6.63$*$	49.35±6.44$*$	46.55±6.73	47.11±7.01$*$	49.49±6.87$*$	49.94±6.74$*$	49.93±6.43$*$	48.58±6.73$*$	**45.66±6.88**
	Housing	50.20±5.92$*$	50.98±4.56$*$	49.58±5.31$*$	50.59±5.41$*$	49.94±5.04$*$	49.39±6.00$*$	48.08±5.40	50.67±5.65$*$	51.60±5.53$*$	53.56±5.19$*$	50.77±5.49$*$	**47.43±5.11**
	Computer	52.71±2.88$*$	57.04±5.06$*$	53.94±4.28$*$	52.42±2.96$*$	54.31±3.62$*$	60.05±4.64$*$	59.62±4.42$*$	52.67±2.99$*$	55.64±4.20$*$	57.23±5.00$*$	52.65±3.18$*$	**49.63±2.72**
	Obesity	67.69±4.79$*$	67.80±6.07$*$	64.22±4.58$*$	64.31±4.81$*$	70.25±4.62$*$	91.30±5.20$*$	86.39±5.84$*$	68.13±4.72$*$	74.40±6.44$*$	83.12±6.31$*$	64.42±4.86$*$	**62.45±4.36**
	Stock	81.81±5.95$*$	78.06±5.91$*$	77.58±6.23$*$	77.32±6.00	83.11±7.66$*$	82.02±7.19$*$	76.96±6.08	81.87±6.25$*$	80.30±6.44$*$	78.89±5.79$*$	78.56±6.35$*$	**75.56±6.32**
	*AvgRank*	7.44	8.11	4.78	4.33	6.56	7.11	7.44	7.89	8.11	10.33	4.56	1.33
AULC-MAE	Newthyroid	7.82±2.85$*$	7.09±3.04$*$	6.44±2.64$*$	6.58±2.73$*$	5.82±3.01	6.72±3.91$*$	6.70±3.42$*$	8.08±2.69$*$	6.24±3.01$*$	7.51±3.09$*$	7.00±2.66$*$	**5.38±2.83**
	Balance-scale	17.80±4.06$*$	17.64±4.00$*$	15.58±3.57$*$	13.94±3.80	14.47±3.47	14.03±2.72	16.52±2.99$*$	18.07±3.96$*$	15.14±3.45	21.45±7.49$*$	**13.56±3.15**	14.09±3.19
	Thyroid	8.66±2.59	16.01±9.26$*$	11.01±5.44$*$	9.20±2.71	10.11±4.13$*$	18.70±10.49$*$	20.52±11.17$*$	8.84±2.50	14.22±8.09$*$	15.64±10.54$*$	**7.81±2.69**$*$	8.52±2.95
	Knowledge	13.49±3.97$*$	13.71±3.95$*$	12.04±3.73	12.30±3.63$*$	12.65±3.83$*$	11.92±3.65	14.69±3.61$*$	13.06±3.79$*$	13.45±3.99$*$	14.24±4.07$*$	12.11±3.48$*$	**11.22±3.20**
	Machine	59.44±8.75$*$	56.19±9.22$*$	57.09±9.93$*$	57.61±9.66$*$	57.61±8.95$*$	54.13±9.37	54.73±9.52$*$	59.33±10.59$*$	57.39±9.30$*$	58.70±9.08$*$	57.63±9.78$*$	**52.78±9.74**
	Housing	60.84±8.75$*$	62.37±7.31$*$	60.94±8.32$*$	61.92±8.50$*$	58.66±7.50$*$	57.94±9.26$*$	57.00±7.99	61.83±9.48$*$	61.68±8.56$*$	64.17±8.05$*$	61.60±8.47$*$	**56.01±7.74**
	Computer	63.84±4.97$*$	70.92±8.64$*$	65.29±6.67$*$	63.71±5.10$*$	65.77±6.92$*$	77.37±10.87$*$	77.36±11.07$*$	63.56±4.84$*$	66.66±7.27$*$	69.96±9.96$*$	63.86±5.37$*$	**59.62±4.62**
	Obesity	90.74±8.06$*$	88.68±8.89$*$	85.57±6.86$*$	85.89±7.39$*$	94.33±7.96$*$	129.51±9.81$*$	118.48±10.88$*$	91.04±7.71$*$	98.52±10.38$*$	111.97±10.23$*$	86.16±7.98$*$	**80.28±6.84**
	Stock	95.15±9.12$*$	88.08±8.66$*$	87.86±8.32$*$	87.51±8.17	97.83±11.82$*$	94.43±9.98$*$	86.59±8.59	95.60±8.98$*$	92.61±9.80$*$	88.60±7.89$*$	89.89±9.18$*$	**84.22±8.01**
	*AvgRank*	7.78	8.11	4.89	4.89	6.33	6.78	7.44	8.22	7.11	9.89	5.11	1.44
AULC-MI	Newthyroid	25.26±6.72$*$	28.79±7.07$*$	29.83±6.77$*$	29.34±6.75$*$	32.88±6.70	**33.79±6.44**$*$	33.46±6.05$*$	24.51±6.56$*$	32.35±6.44$*$	26.89±7.05$*$	28.21±6.31$*$	33.52±6.87
	Balance-scale	26.53±4.87$*$	30.23±4.64$*$	31.12±4.11$*$	31.73±4.95	31.13±4.51	31.75±3.51	28.85±4.01$*$	26.17±4.89$*$	30.05±4.27	23.80±7.95$*$	**32.06±4.18**	30.87±4.34
	Thyroid	3.65±1.18	3.53±1.00$*$	4.25±0.94$*$	3.79±1.04	4.10±1.01$*$	3.33±1.01$*$	3.51±0.87$*$	3.99±1.16	3.57±0.87$*$	2.78±1.36$*$	**4.49±1.07**$*$	4.42±0.96
	Knowledge	72.22±8.42$*$	73.06±7.75$*$	75.73±8.20	75.17±7.77$*$	74.16±8.56$*$	75.89±8.25	71.25±6.42$*$	73.33±7.85$*$	72.37±8.50$*$	72.89±7.20$*$	75.39±7.55$*$	**76.78±7.25**
	Machine	64.20±8.35$*$	68.21±9.50$*$	66.78±9.54$*$	66.43±9.19$*$	65.92±8.63$*$	71.44±9.00	70.21±9.39$*$	65.30±10.00$*$	66.42±9.57$*$	67.38±9.16$*$	66.08±8.89$*$	**72.59±9.42**
	Housing	55.52±8.40$*$	53.66±6.96$*$	55.27±8.29$*$	54.63±7.99$*$	58.53±7.57$*$	61.35±7.95$*$	61.33±7.69	54.76±8.57$*$	55.43±8.34$*$	53.51±8.17$*$	54.71±8.39$*$	**61.44±7.74**
	Computer	47.50±4.59$*$	43.37±7.13$*$	47.01±5.66$*$	47.84±4.42$*$	46.71±6.01$*$	41.42±6.91$*$	41.77±6.63$*$	47.91±4.29$*$	46.55±6.18$*$	44.84±7.93$*$	47.68±5.14$*$	**52.80±4.14**
	Obesity	129.63±8.26$*$	130.69±9.86$*$	135.04±8.24$*$	134.62±7.64$*$	127.17±7.43$*$	92.56±8.44$*$	101.46±9.19$*$	130.41±8.21$*$	120.20±9.67$*$	108.64±8.84$*$	134.93±7.98$*$	**137.76±8.08**
	Stock	280.21±9.53$*$	286.94±10.07$*$	288.01±10.33$*$	288.72±10.24	278.42±11.86$*$	281.62±10.16$*$	289.16±10.35	279.56±9.87$*$	282.99±11.14$*$	287.85±9.18$*$	286.57±10.65$*$	**293.74±9.67**
	*AvgRank*	8.67	7.56	4.67	5.33	6.56	5.89	7.11	8.11	7.78	9.33	5.22	1.78

**Table 3:** Win/tie/loss counts of the proposed method versus the baseline methods based on the Wilcoxon signed-rank test at a 5% confidence level.

	Random	USME	USLC	USMS	MCSVMA	McPAL	iGS	FISTA	ALCE	LogitA	ALOR
AULC-MZE	8/1/0	9/0/0	8/1/0	6/3/0	7/2/0	6/3/0	7/2/0	8/1/0	8/1/0	9/0/0	7/1/1
AULC-MAE	8/1/0	9/0/0	8/1/0	6/3/0	7/2/0	6/3/0	7/2/0	8/1/0	8/1/0	9/0/0	7/1/1
AULC-MI	8/1/0	9/0/0	7/1/1	6/3/0	7/2/0	5/3/1	7/2/0	8/1/0	8/1/0	9/0/0	7/1/1

The results in Table 2 show that the proposed method performs better than the competitors on most datasets in terms of the metrics AULC-MZE, AULC-MAE, and AULC-MI, respectively. Although the AOCECM does not perform best on some of the data, the results of the Wilcoxon test in Table 3 show that the AOCECM significantly outperforms most of the compared methods on most datasets. Furthermore, the results of the average ranks in Table 2 show the proposed method is among the top performers.

In the compared methods, USME, USLC, and USMS are three different uncertainty sampling strategies. We instantiate these strategies based on the KELMOR model. The USME selects the query instance with the highest information entropy. The USLC queries the instance with the lowest maximum in predictions over all classes. The USMS queries the unlabeled instance with the lowest discrepancy in its top two class predictions. From Table 2, we can see that USLC and USMS perform better than USME. The performances of USMS are comparable to USLC on the metric AULC-MAE, but USMS performs better on the metric AULC-MZE. In ordinal data, the informative instances are usually distributed in the regions between adjacent classes. The margin-based sampling criterion in USMS tends to query instances in those regions. Therefore, our method incorporates the margin-based sampling criterion with the expected cost minimization criterion. This combination imposes our method to select query instances from those informative regions that can reduce the KELMOR model’s misclassification cost.

The method MCSVMA selects instances based on rejection, compatibility, and uncertainty criteria. However, these criteria are designed based on an SVM model with the one-versus-rest scheme. Therefore, this method is more suitable for nominal multi-class classification problems rather than ordinal classification problems. McPAL also only considers the nominal multi-class classification settings. Therefore, its performance on ordinal data is inferior to the proposed method. The method iGS is an AL method for regression problems. This method performs query selection by considering the diversity of both input and output spaces. However, since this method relies on a regression model, it cannot capture informative instances in ordinal data. FISTA is a transductive experimental design-based method that queries representative unlabeled instances based on a data reconstruction mechanism. Since it does not rely on a prediction model, it failed to consider the informativeness of the query instances. ALCE performs query selection based on a cost-embedding uncertainty criterion. Since this approach tends to select the instances with the highest misclassification cost in the current prediction model, this approach is susceptible to sampling bias in the ordinal classification setting. Although the method LogitA is designed for ordinal classification, the overall performance of LogitA is not well. This is because the A-optimal experimental design-based criterion tends to query representative instances but fails to select the discriminative ones. The ALOR method performs query selection based on a threshold-based ordinal classification model and a margin-based sampling criterion. This method selects the informative instances distributed between adjacent classes and performs similarly to the USMS. However, there is no mechanism to maintain the diversity of the selected instances, which leads to this method suffering from sampling redundancy. Multiple factors bring the outstanding performance of the proposed method. On the one hand, we simultaneously consider the ordering information and the margin-based uncertainty criterion, ensuring our method selects more informative instances. On the other hand, the *k*-means clustering-based candidate instance selection ensures the selected instances have the properties of representative and diversity. This makes the critical instances selection more effective.

The proposed method integrates the expected cost minimization criterion and the margin sampling criterion with a trade-off parameter $\lambda$. To examine which criterion is more important and how to set the value of $\lambda$, we set $\lambda = [0.1,0.2,\ldots ,1.0]$ and record the average rank of the AOCECM methods with different $\lambda$ values on the metrics AULC-MZE, AULC-MAE, and AULC-MI, respectively. We present the results of the average rank in Table 4. From the results, we can see that the appropriate values of $\lambda$ concerning MZE, MAE, and MI are 0.7, 0.9, and 1.0, respectively. Since ordinal classification focuses more on the evaluation metric MAE, we recommend setting the value of $\lambda$ to 0.9 or a relatively large value in practice. The results illustrate that the expected cost minimization criterion is more important. Although the average rank results with $\lambda =0.9$ are close to that with $\lambda =1.0$, it does not indicate the margin-based sampling has no contribution to our algorithm because, on most datasets, the participation of margin-based sampling in our algorithm brings a positive impact on the results. However, how to adaptively determine the optimal value of $\lambda$ is a problem that needs further study.

**Figure 6:**
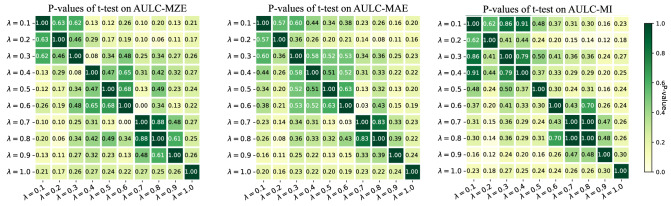
*P*-values of paired t-tests between the AOCECM methods with different $\lambda$ values on the metrics AULC-MZE, AULC-MAE, and AULC-MI, respectively.

To examine whether the AOCECM method is sensitive to parameter $\lambda$, we conduct the paired t-test between the AOCECM methods with different $\lambda$ values at a confidence level of 0.05. We show the p-values of the paired t-tests on metrics AULC-MZE, AULC-MAE, and AULC-MI in Fig. 6. We can see that the p-values in the three sub-figures are larger than 0.05 in most cases. Therefore, the proposed method is almost insensitive to the parameter $\lambda$.

**Table 4:** Average rank of the AOCECM methods with different $\lambda$ values on the metrics AULC-MZE, AULC-MAE, and AULC-MI.

Metric	$\lambda = 0.1$	$\lambda = 0.2$	$\lambda = 0.3$	$\lambda = 0.4$	$\lambda = 0.5$	$\lambda = 0.6$	$\lambda = 0.7$	$\lambda = 0.8$	$\lambda = 0.9$	$\lambda = 1.0$
AULC-MZE	6.78	6.89	7.22	5.67	5.56	5.67	**4.22**	4.33	4.33	4.33
AULC-MAE	5.89	7.22	6.56	5.89	6.11	5.56	4.67	4.67	**3.78**	4.67
AULC-MI	5.22	5.89	5.89	6.56	6.22	6.00	5.33	6.33	4.11	**3.44**

Execution time is an important concern for active learning methods. Therefore, the average time consumption of the different methods by performing 20*K* query selections on the nine datasets was recorded and summarized in Table 5. We do not show the time consumption of the random sampling method (Random) because its time consumption is almost negligible. In Table 5, the AOCECM$*$ is the method AOCECM without candidate subset selection. We can see that the time consumption of AOCECM is significantly lower than that of AOCECM$*$. This illustrates that the candidate subset selection is effective in reducing the computational burden of AOCECM.

**Table 5:** Execution time (second) of different methods.

Datasets	USME	USLC	USMS	MCSVMA	McPAL	iGS	FISTA	ALCE	LogitA	ALOR	AOCECM	AOCECM$*$
Newthyroid	0.10	0.06	0.12	0.17	0.56	0.04	0.11	3.38	1.23	0.24	1.17	21.94
Balance-scale	0.23	0.07	0.22	0.19	2.00	0.07	0.44	3.54	2.91	0.58	2.88	108.90
Thyroid	2.78	0.75	2.42	1.55	43.39	0.85	40.11	5.89	40.89	5.85	15.67	9539.56
Knowledge	0.26	0.08	0.21	0.31	2.19	0.09	0.26	6.66	3.44	0.77	4.70	104.18
Machine	0.20	0.10	0.14	0.32	1.23	0.09	0.10	10.66	2.58	0.74	4.11	44.13
Housing	0.44	0.12	0.23	0.58	4.19	0.13	0.34	11.14	18.69	1.81	9.53	195.31
Computer	7.38	1.53	3.72	8.94	118.06	1.70	54.26	21.13	299.27	23.90	105.43	30125.89
Obesity	3.31	0.57	1.33	4.24	44.04	0.57	3.02	28.46	268.03	21.60	114.69	4923.63
Stock	2.62	0.49	0.93	3.49	45.44	0.39	0.78	54.87	124.53	43.57	139.82	2611.01

## Conclusion and future work

This paper studies the problem of active learning for ordinal classification. The present study innovatively takes the ordering information into account in query selection by designing an expected cost minimization criterion. To fully use the available information, we integrate the expected cost minimization with the margin-based uncertainty sampling criterion to select query instances in a complementary way. Considering the computationally intensive of calculating the expected cost, we make it tractable by introducing a *k*-means clustering-based candidate subset selection method. This method substantially reduces the computational overhead of our algorithm and endows the query instances with the properties of representative and diversity. Extensive experiments on nine public datasets demonstrate that the proposed AL method can achieve better performance than the competitors.

The following four works merit further investigation: (1) It is interesting and practical to consider the misclassification and labeling costs simultaneously. Therefore, proposing a cost-sensitive AL method to learn a promising ordinal classifier with minimal comprehensive cost is worthwhile. (2) To further reduce the labeling cost, we would like to consider the annotator can provide low-cost instance-pair relation information^11^. Thus, investigating active learning for ordinal classification by querying instance-pair relation information is valuable. (3) In practice, we cannot guarantee that the annotators can always provide the ground-truth labels. Therefore, it is interesting to investigate an active ordinal classification method that can use noisy labeling sources^62,63^. (4) Ordinal classification problems in many fields may involve image data. Therefore, extending the proposed method to the convolutional neural networks is valuable for implementing active learning on image ordinal data.

## Data Availability

The datasets used in this study are available at https://github.com/DeniuHe/AOCECM.
